# Spatial Modeling of Douglas‐Fir Plantations in Italy After 120 Years of Experimentation

**DOI:** 10.1002/ece3.71943

**Published:** 2025-08-07

**Authors:** Maurizio Marchi, Martina Cocozza, Gabriele Bucci, Paolo Iovieno

**Affiliations:** ^1^ CNR‐Institute of Biosciences and BioResources, Florence Research Area Sesto Fiorentino Italy

**Keywords:** climate, ecological modeling, exotic tree species, forest tree provenances, habitat suitability

## Abstract

This study aims to identify the ecological factors that drive the survival of Douglas‐fir ( *Pseudotsuga menziesii* [Mirb.] Franco) in Italy, using data from old‐growth experimental stands. A record of 124 Douglas‐fir plantations was compiled from a literature review and ground survey, including 98 Douglas‐fir stands established in the early 20th century. The probability of survival of the species at the surveyed sites was modeled using Species Distribution Models (SDM) with soil and climatic variables as predictors. Pseudo‐absences were also used to balance the proportion of presences and absences in the modeling steps. The best‐fitting models were used to predict the probability of survival of Douglas‐fir stands across the entire country and in the native range of the species to assess the model's goodness of fit. Fitted models performed well with a mean True Skill Statistics (TSS) score of 0.91, suggesting that temperature‐related factors primarily influence the survival of Douglas‐fir stands in Italy. The two most relevant predictors were GDD5 (growing degree‐days above 5°C) and AHM (Annual Heat Moisture), indicating the importance of temperatures and water availability also in the Mediterranean area. A large portion of Italy was predicted to be potentially suitable for Douglas‐fir afforestation or reforestation, mainly across the Apennine Mountains of central Italy. Model projections for the species' native area largely overlap with the range of the coastal variety of Douglas‐fir ( *P. menziesii var. viridis* ), supporting the hypothesis that most Douglas‐fir stands in Italy were established using propagation material from this region.

## Graphical abstract

The spatial modeling of Douglas‐fir old‐growth stands in Italy demonstrated the relevance of temperatures (GDD5) also in the Mediterranean area and showed a good climate matching between the Italian planting range and the coastal part of Douglas‐fir native range.

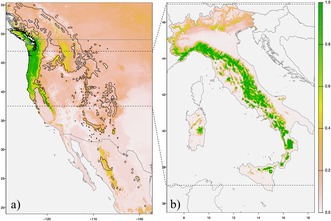

**Abbreviations**
AHM: Annual Heat moisture
ANN: Artificial Neural Networks
AUC: Area Under the receiver operating characteristic (ROC) Curve
CMD: Hargreave's Climatic Moisture Meficit
CTA: Classification Trees Analysis
ENM: Ecological Niche Models
GAM: Generalized Additive Model
GBM: Generalized Boosted Regression Model
GDD5: Growing Degree‐Days above 5°C
GLM: Generalized Linear Model
INFC: Italian National Forest Inventory
MARS: Multivariate Adaptive Regression Splines
MAXENT: Maximum ENTropy
MCMT: Mean Coldest Month Temperature
MGSP: Mean Growing Season Precipitation
RF: Random Forest
SDM: Species Distribution Models
SHM: Summer Heat moisture
TD: Temperature Difference
TSS: True Skill Statistics
XGBOOST: eXtreme Gradient BOOSTing

## 1 Introduction

Douglas‐fir ( *Pseudotsuga menziesii* (Mirb.) Franco) is the most widely distributed conifer species in North America and one of the most successful non‐native tree species in Europe. In its native range, Douglas‐fir covers approximately 14.4 million hectares in the USA and 4.5 million hectares in Canada (Wang, Campbell, et al. 2012). Two varieties of the species are identified based on morphological, physiological, and chemical differences. The “*viridis*” variety grows in the Pacific Northwest coastal regions of Canada and the USA, while the “*glauca*” variety occurs in the interior and along the Rocky Mountains from Alberta to Arizona and Colorado (van Loo et al. 2015). Additionally, a distinct Mexican genetic lineage was also identified through chloroplast DNA sequencing (Wei et al. 2011), though its distinctiveness is still a topic of debate. The species is well‐adapted to a wide range of climatic conditions throughout its extensive distribution, which spans from oceanic to continental climates. Research has indicated that Douglas‐fir is drought‐resistant (Bansal et al. 2015; Leuschner and Meinzer 2024), tolerant to various pathogens (Hawkins and Henkel 2011), and exhibits efficient water usage and high biomass production (Smolnikar et al. 2021). These ecological characteristics have led to the extensive cultivation of Douglas‐fir for timber and pulp, making it suitable for afforestation/reforestation activities in Europe as well. Currently, Douglas‐fir plantations cover approximately 830,000 ha, primarily in France (50.6%) and Germany (26.5%). It is regarded as a sustainable choice in the context of future climate change scenarios (Frei et al. 2022). This is particularly relevant in Europe, where *Picea abies* plantations suffer from phytosanitary problems (e.g., massive attacks of *Ips typographus*) or maladaptation (Vitali et al. 2017).

In the 1960s, a joint experimental program was launched in Europe on Douglas‐fir under the supervision of the International Union of Forest Research Organizations (IUFRO). About 182 provenances of Douglas‐fir were tested, collecting seeds from almost all the native range, which were planted in common gardens in 36 countries worldwide, including 15 European countries (Alizoti et al. 2022; Bastien et al. 2013). The provenances from the Northwestern United States (Washington State and Oregon) showed the best performance in the European planting sites (Isaac‐Renton et al. 2014). More recently, several studies have focused on the origin of the material used for current Douglas‐fir plantations in Europe (van Loo et al. 2019), to investigate their ecological performance in different European stands (Castaldi et al. 2017; Hintsteiner et al. 2018), or to develop transfer models under different climate change scenarios (Chakraborty et al. 2018). In this framework, Italy represents the southernmost case study for Douglas‐fir plantations in Europe. According to the literature (De Rogatis et al. 2024; Marchi and Cocozza 2021), three IUFRO test trials and 98 experimental trials were established across the Italian Peninsula, most of which were formerly studied by Prof. Aldo Pavari in the early 1900s. These trials/experimental sites provided unsatisfactory results for Douglas‐fir plantations across the Alpine region due to freezing winters and harsh climates, while good growth performances were observed in the Apennine and Mediterranean regions (Castaldi et al. 2017). Of the original 98 experimental plots, 78 were still active in 1941, and only 37 remained by 1981.

Species distribution models (SDMs) and ecological niche models (ENMs) are widely used to examine the relative importance of environmental gradients in the spatial distribution of plant and animal species (Anderson 2017; Booth 2018; Pecchi et al. 2019). Since the early 2000s, predicting changes in the spatial distribution of target species has improved due to the availability of accessible climatic data, occurrence records such as GBIF, forest ecotypes, continental datasets, and national inventories (Booth 2014; Marchi 2023; Mauri et al. 2017; Zhao et al. 2023). The most utilized modeling algorithms include Artificial Neural Networks (ANN), Random Forests (RF), Generalized Linear Models (GLM), Generalized Additive Models (GAM), and the Maximum Entropy model (MaxEnt), with the latter being the most frequently employed in recent studies (Naimi and Araújo 2016; Pecchi et al. 2019; Peterson and Soberón 2012; Qiao et al. 2015; Wiens et al. 2009). A key aspect of SDMs and ENMs is accurately identifying ecological predictors, which can be achieved through an iterative method (Cobos et al. 2019) or by reviewing existing literature on similar case studies. Previous studies on Douglas‐fir in Europe and North America have included key Mediterranean parameters such as drought indices, growing degree‐days above 5°C, and summer heat moisture deficit, alongside classic predictors like mean annual precipitation and temperature (Chakraborty et al. 2018; Flower et al. 2013; Isaac‐Renton et al. 2014).

In this study, we examined the climatic characteristics of habitats that host the surviving Douglas‐fir plantations in Italy. Our primary objective was to identify the key factors driving the species' survival in the Mediterranean climate and delineate the most suitable areas for Douglas‐fir plantations across the country. A comprehensive assessment of the climatological requirements for Douglas‐fir plantations in Italy is still lacking, although some local attempts have been made in the past.

## 2 Materials and Methods

To identify the primary ecological drivers of Douglas‐fir survival in Italy, two datasets were combined within a modeling framework. The first dataset was obtained by reconstructing the geographic distribution of the experimental plots established by Aldo Pavari between 1921 and 1940 across the Alps, along the entire Apennine chain, and on the two major islands, Sardinia and Sicily. The second dataset was a climatological dataset tailored to the age of each stand, based on the planting year and the survival of the trees at each site.

### 2.1 Spatial Occurrences Dataset

We combined four data sources on the Italian Douglas‐fir stands containing data since 1941: (i) the forest inventories carried out by the Forestry Experimental Institute of the Italian Ministry of Agriculture in 1940 and 1980 on the aforementioned 98 experimental plots; (ii) the data recently published by Castaldi et al. (2017) on 20 Douglas‐fir stands in Central Italy; (iii) the Italian National Inventory (INFC2015) reporting the species' presence in 45 plots; and (iv) the six stands listed in the Italian National Register of Forest Basic Materials. Some of the stands were documented by multiple sources mentioned above. When necessary, we integrated the collected information by visiting the sites to verify the survival of Douglas‐fir trees.

According to Avolio and Bernardini (2000), the 98 experimental plantations made between the early 1900s and 1933 were inventoried in 1940 and 1980, and the relative data are currently available as aggregated records (i.e., no tree‐level data available). All the inventoried stands had limited extension (often < 1 ha), with trees planted in rows with a spacing between 2 and 3 m. In a few cases, approximate information about the seed/seedling provenance used in plantations was recorded (e.g., “British Columbia,” “Washington State,” or “Seattle area”), whereas for most stands, the geographic coordinates and elevation of the planting sites were missing (Pavari and De Philippis 1941). Therefore, the exact location and current existence of each non‐georeferenced Douglas‐fir plantation were determined by visiting these sites during the spring and summer of 2024. Overall, we could reconstruct the history of 78 out of the 98 Pavari's experimental sites, where Douglas‐fir was still present in 1940, 1980, and 2024. All the other records (i.e., Castaldi et al. 2017, INFC2015, and the National Register of Forest Basic Material) had geographic coordinates and measurement parameters (volumes, basal area, diameters, and height), and the survival of Douglas‐fir was confirmed by visiting the corresponding sites in spring and summer 2024.

**FIGURE 1:**
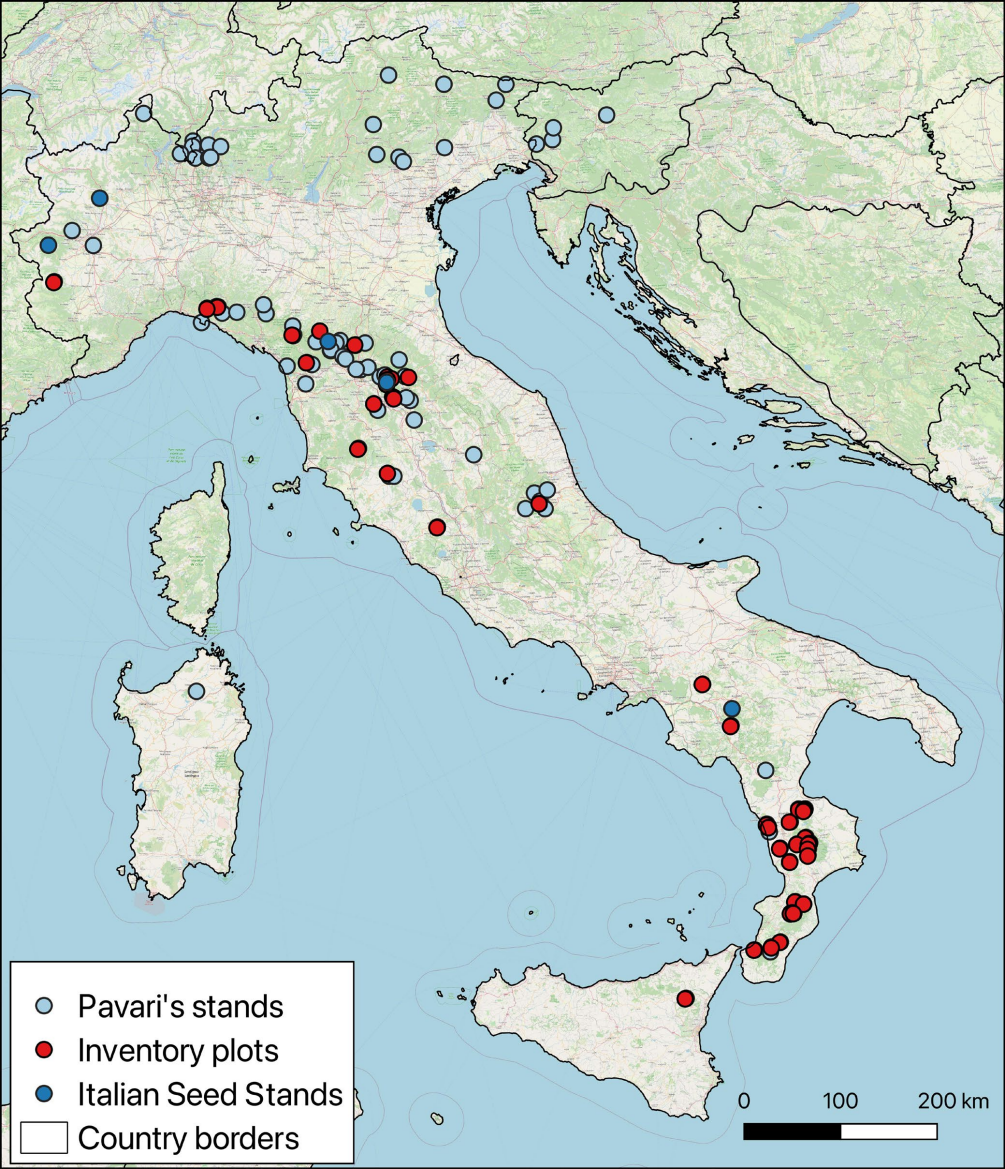
Spatial distribution of the Italian Douglas‐fir plantations included in this study. Light blue points represent Pavari's experimental stands; red points represent INFC2015 National inventory points; blue points are the stands included in the Italian National Register of Forest Basic Materials (MASAF and the FOREMATIS database). The data are plotted over the OpenStreet Map layer.

Combining the above information, we mapped 124 Douglas‐fir plantations spread across Italy (Figure 1). For each stand, the presence of Douglas‐fir over time (1940, 1980, 2015, and 2024) was documented, based on the aforementioned sources and on‐site visits, along with the year of planting (when available). This allowed us to infer the survival of Douglas‐fir plantations at each site over time.

### 2.2 Climatic Dataset and Soil Data

The climatic information was obtained from the ClimateDT web portal (Marchi et al. 2024), available at https://www.ibbr.cnr.it/climate‐dt, an open‐source, scale‐free downscaling tool where climatic data at a global scale since 1901 are freely downloadable as monthly time series. ClimateDT can generate more than 90 ecologically relevant climatic indices and variables through statistical downscaling with local adjustment for elevation. It also includes future scenarios from the Fifth and the Sixth Assessment Report (AR5 and AR6) based on CHELSA (Karger et al. 2017) and CRU‐TS layers (Harris et al. 2020). Using the geographic coordinates and elevation of the above‐mentioned 124 Douglas‐fir stands as inputs for ClimateDT, we obtained the complete climatological series (1901–2024) at each site. For each stand, we averaged local climatic parameters over the period from the date of establishment (inferred from available documents) to the date of the last inventory where the stand was included (i.e., the stand was still alive). For example, for all the plantations that died before the first inventory period (i.e., before 1940), the average climatic parameters between the plot establishment and 1940 were calculated. As for the plots that survived up to the second inventory (1980) or are currently on site (2024), we computed a 30‐year normal climate using the last survey year as the last year of the climatic data series. For stands reported as still existing in 1940, 1980, or 2015 but not detected in 2024, we relied on the information available from the last inventory that confirmed their existence, as it was not possible to determine whether their absence in 2024 was due to maladaptation, biotic stress, or harvesting. The rasters of the last climatological normal period (i.e., the average climate across 30 years, in this case 1991–2020) were also generated by ClimateDT and used for the spatial predictions of habitat suitability across Italy, while for testing the model performances over the species' native range, the average climatic series of 1901–1930 was considered, as the propagation material used for the Italian plantations dates back to that period.

We selected seven biologically meaningful climate variables and indices to account for most of the climatic variance across the study area, namely: (i) Annual Heat Moisture (AHM), (ii) Hargreave's climatic moisture deficit (CMD), (iii) the growing degree‐days above 5°C (GDD5), (iv) Mean Coldest Month Temperature (MCMT), (v) Mean Growing Season Precipitation (MGSP), (vi) Summer Heat Moisture (SHM), and (vii) Temperature Difference (TD). These variables, their ecological meaning, and the methods used for calculation from time series and standard variables are explained in detail in Marchi et al. (2024) and Wang, Hamann, et al. (2012). Additionally, we extracted soil properties and characteristics at each Douglas‐fir stand site from the soil database developed at the Soil Cartography Laboratory of the Council for Agricultural Research and Economics (CREA, Italy), which is accessible through the Zenodo website (Costantini et al. 2014). This data source has already been used in a similar study on poplar stands (Corona et al. 2024) and includes several soil properties of interest, such as soil depth, active carbonates, pH in water, soil salinity, soil texture, and soil drainage.

### 2.3 Data Analysis

Both climatic and soil data from the 124 stand sites were used as input data in a framework where Douglas‐fir survival (i.e., 1 = survival, 0 = death) was modeled as a function of local climatic drivers. After reviewing the literature and considering various modeling techniques, we chose to use the R package “biomod2” Ensemble Platform for Species Distribution Modeling (Thuiller et al. 2023). This tool includes 10 modeling algorithms used to build an Ensemble mean model, weighting the performance of each algorithm using several statistics such as the Area Under the Receiver Operating Characteristic curve (AUC or ROC, Pecchi et al. 2019), the True Skill Statistics (TSS, Leroy et al. 2018), and several other metrics available in the biomod2 package. The tested algorithms were ANN, Classification Trees Analysis (CTA), GAM, Generalized Boosted Regression Model (GBM), GLM, Multivariate Adaptive Regression Splines (MARS), Maximum entropy (MAXENT), RF, and Extreme Gradient Boosting (XGBOOST).

Given the unbalanced distribution of occurrences in the spatial dataset (83 verified presences of living Douglas‐fir stands and 41 true absences due to maladaptation at the 124 study sites), we used the internal biomod2 functions to generate pseudo‐absences. According to Barbet‐Massin et al. (2012), 10 pseudo‐absence datasets were generated, and a total of 410 pseudo‐absences were added to the spatial dataset. Occurrences were checked for spatial autocorrelation prior to modeling, and all algorithms were built by randomly splitting the data into calibration and validation datasets, each containing 75% and 25% of the data, respectively. The random splitting of the data was repeated 100 times, and the model scores were averaged over runs. The best‐performing models were then used to build a consensus Ensemble habitat suitability map for Douglas‐fir stands over the Italian territory.

Once models were fitted, a spatial projection was done in Italy using the 1991–2020 normal climate to map the potential areas of suitability of the species. A parallel projection was also done in the native range (i.e., the Pacific Northwest of the USA) using the 1901–1930 normal climate to match the climatic characteristics existing in the native range at the time the propagation material of the Italian stands was collected. This “reverse projection” was done to test the accuracy of the model and to have a degree of climatic similarities between the Italian sites and different parts of the species' native range. Finally, a multivariate cluster analysis of the environmental characteristics of the Italian Douglas‐fir sites was conducted by selecting a subset (35 stands) of the 124 study sites to minimize possible redundancy resulting from similar climatic characteristics in geographically close stands. All 46 climatic parameters obtained from ClimateDT were standardized by rescaling to a mean of 0 and a standard deviation of 1 and then used to build the dendrogram. The final dendrogram was generated using the Euclidean distance matrix among the stands and a “complete” aggregation method.

All the models, analyses, and results were run and generated using R Statistical Software (R Core Team 2025).

## 3 Results

In this study, we examined the relative importance of seven selected climatic variables and six soil variables as potential predictors of Douglas‐fir survival in 124 locations across Italy. The results showed that all soil properties were poor predictors of Douglas‐fir survival and were discarded from further analysis, while only the seven climatic variables were used to train the final model. Table 1 reports the average values of evaluation metrics and their standard deviations for different runs of the model using the training dataset. The biomod2 modeling revealed that the RF technique was the most effective algorithm, scoring higher in 10 out of the 12 metrics utilized for data fitting assessment. The full ranking list also reported GBM, GAM, and XGBOOST algorithms as very well‐performing and worthy of mention. In contrast, ANN and CTA were often less powerful, and MAXENT was heavily dependent on the evaluation metric. On average, RF exhibited TSS values of 0.85 and ROC of 0.93. Only the neural network algorithm (ANN) showed evaluation metrics that were often below the average and was therefore excluded from the Ensemble modeling.

**TABLE 1:** Fitted models with biomod2 ensemble forecasting platforms (columns) and their fitting evaluation metrics (rows) obtained across 10 PA datasets and 54 runs of repetitions.

		ANN	CTA	GAM	GBM	GLM	MARS	MAXENT	RF	XGBOOST	AVERAGE
ACCURACY	Mean	0.793	0.886	0.921	0.925	0.906	0.917	0.915	**0.933**	0.911	0.901
	StDev	0.079	0.042	0.033	0.035	0.039	0.038	0.037	**0.031**	0.037	0.041
BIAS	Mean	0.809	0.899	0.920	**0.922**	0.913	0.916	0.910	0.921	0.910	0.902
	StDev	0.250	0.086	0.064	**0.066**	0.073	0.070	0.072	**0.066**	0.071	0.091
CSI	Mean	0.624	0.761	0.826	0.832	0.798	0.817	0.816	**0.842**	0.803	0.791
	StDev	0.104	0.072	**0.066**	0.072	0.074	0.075	0.071	**0.066**	0.073	0.075
ETS	Mean	0.416	0.627	0.724	0.736	0.682	0.712	0.708	**0.751**	0.692	0.672
	StDev	0.155	0.108	0.100	0.108	0.109	0.111	0.108	**0.099**	0.107	0.112
FAR	Mean	0.810	0.859	0.887	0.963	0.910	0.923	0.883	**0.974**	0.956	0.907
	StDev	0.153	0.082	0.050	0.043	0.071	0.067	0.064	**0.034**	0.047	0.068
KAPPA	Mean	0.569	0.765	0.836	0.843	0.806	0.827	0.824	**0.854**	0.813	0.793
	StDev	0.177	0.083	0.069	0.075	0.080	0.078	0.075	**0.066**	0.077	0.087
ORSS	Mean	0.899	0.943	0.985	0.986	0.980	0.981	0.983	**0.990**	0.979	0.970
	StDev	0.022	0.019	0.020	0.016	0.024	0.023	0.018	**0.011**	0.024	0.020
POD	Mean	0.981	0.945	0.972	0.959	0.946	0.943	0.916	**0.974**	0.955	0.954
	StDev	0.034	0.051	0.039	0.040	0.060	0.059	0.060	**0.030**	0.046	0.046
POFD	Mean	0.997	**0.999**	0.988	0.978	0.960	0.962	0.975	0.986	0.973	0.980
	StDev	0.012	**0.010**	0.023	0.026	0.050	0.048	0.049	0.020	0.033	0.030
ROC	Mean	0.854	0.896	0.961	0.971	0.943	0.948	0.915	**0.977**	0.957	0.936
	StDev	0.109	0.046	0.025	0.021	0.042	0.037	0.037	**0.017**	0.025	0.040
SR	Mean	0.810	0.859	0.910	0.963	0.910	0.923	0.883	**0.974**	0.887	0.902
	StDev	0.153	0.082	0.099	0.043	0.071	0.067	0.064	**0.034**	0.054	0.074
TSS	Mean	0.589	0.770	0.838	0.846	0.812	0.831	0.830	**0.855**	0.816	0.799
	StDev	0.179	0.082	0.069	0.073	0.078	0.078	0.074	**0.066**	0.076	0.086

*Note:* The best‐performing algorithms, which showed the highest mean value and the lowest standard deviation, are highlighted in bold.

**FIGURE 2:**
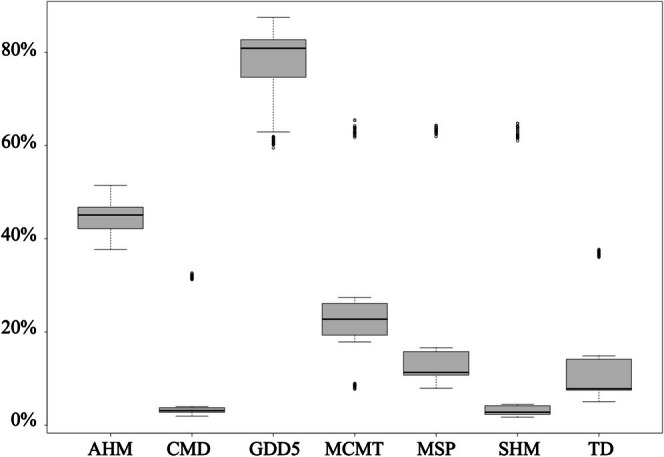
Relative variable importance of the seven climatic predictors included in the ensemble model. The box plots represent the relative importance of each predictor, as indicated by the proportion of variance explained in the model (*y*‐axis—the higher the value, the greater the variable's influence on the model). Data obtained after 2450 permutation tests. Note that this analysis does not account for interactions between the variables.

**FIGURE 3:**
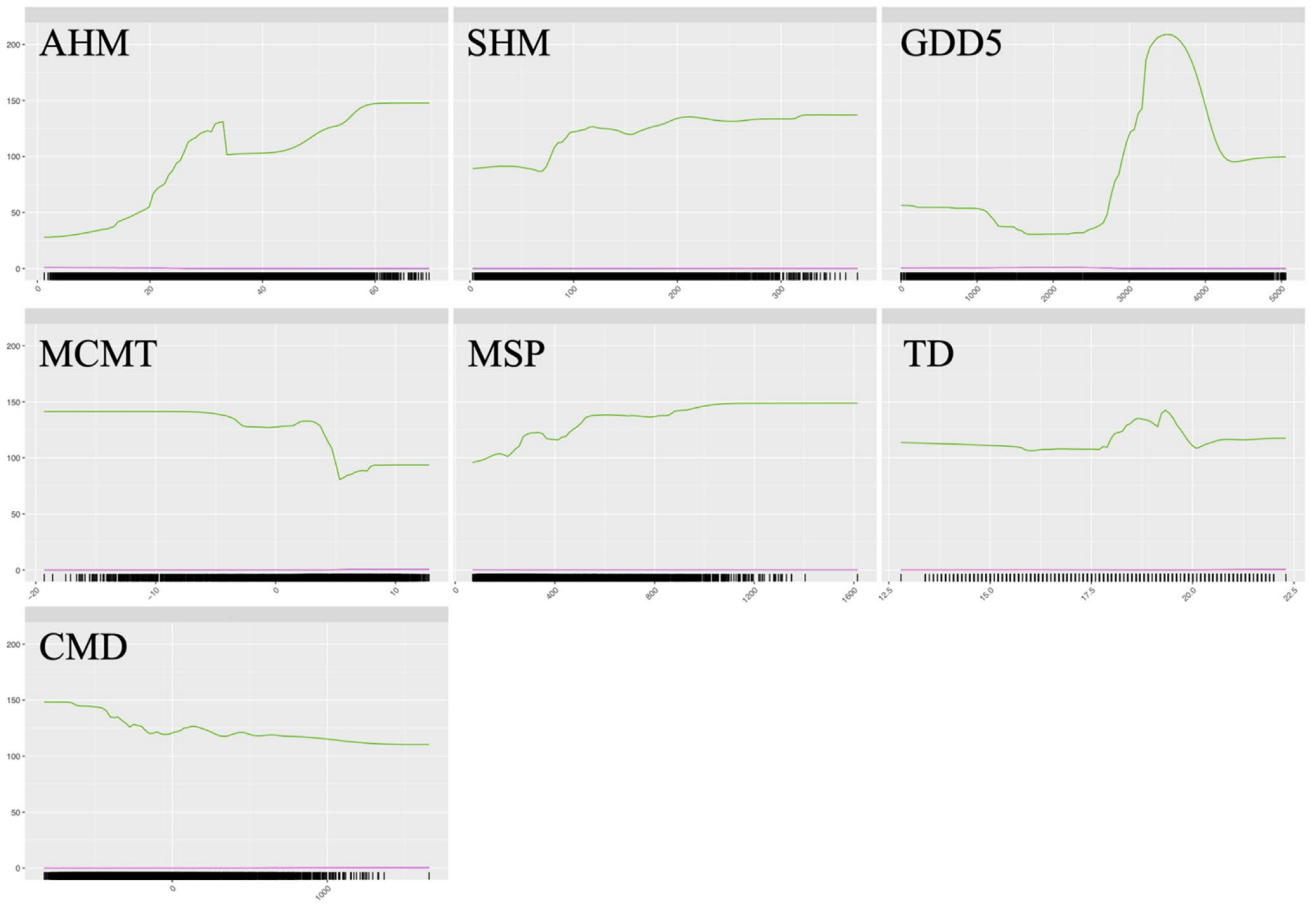
Results of the sensitivity analysis of the seven climatic variables used in modeling the survival of Douglas‐fir stands across Italy. The response curves show the model's sensitivity to the bioclimatic variables, with the probability of survival of the species on the *y*‐axis and the environmental variable on the *x*‐axis. Note that this analysis does not account for interactions between the variables.

The contribution of each variable in the model and the sensitivity analysis across its range of variation were obtained from the ensemble model as the weighted mean of the best‐performing algorithms. The GDD5 and AHM predictors had the highest importance (Figure 2). Under the permutation tests, the variability of the prediction associated with GDD5 was approximately 80%, while AHM showed values around 42%. Conversely, SHM and CMD were less relevant after excluding outliers. Notably, a large variance was observed for all the predictors, except for CMD and SHM, across different models and datasets. The sensitivity analysis also confirmed the above findings, with GDD5 and AHM showing a clear pattern across their range of variation. In contrast, the curves for the other variables, especially CMD and SHM, appeared much flatter and, therefore, less relevant to characterize the probability of survival of the target species across the investigated ecological range (Figure 3).

**FIGURE 4:**
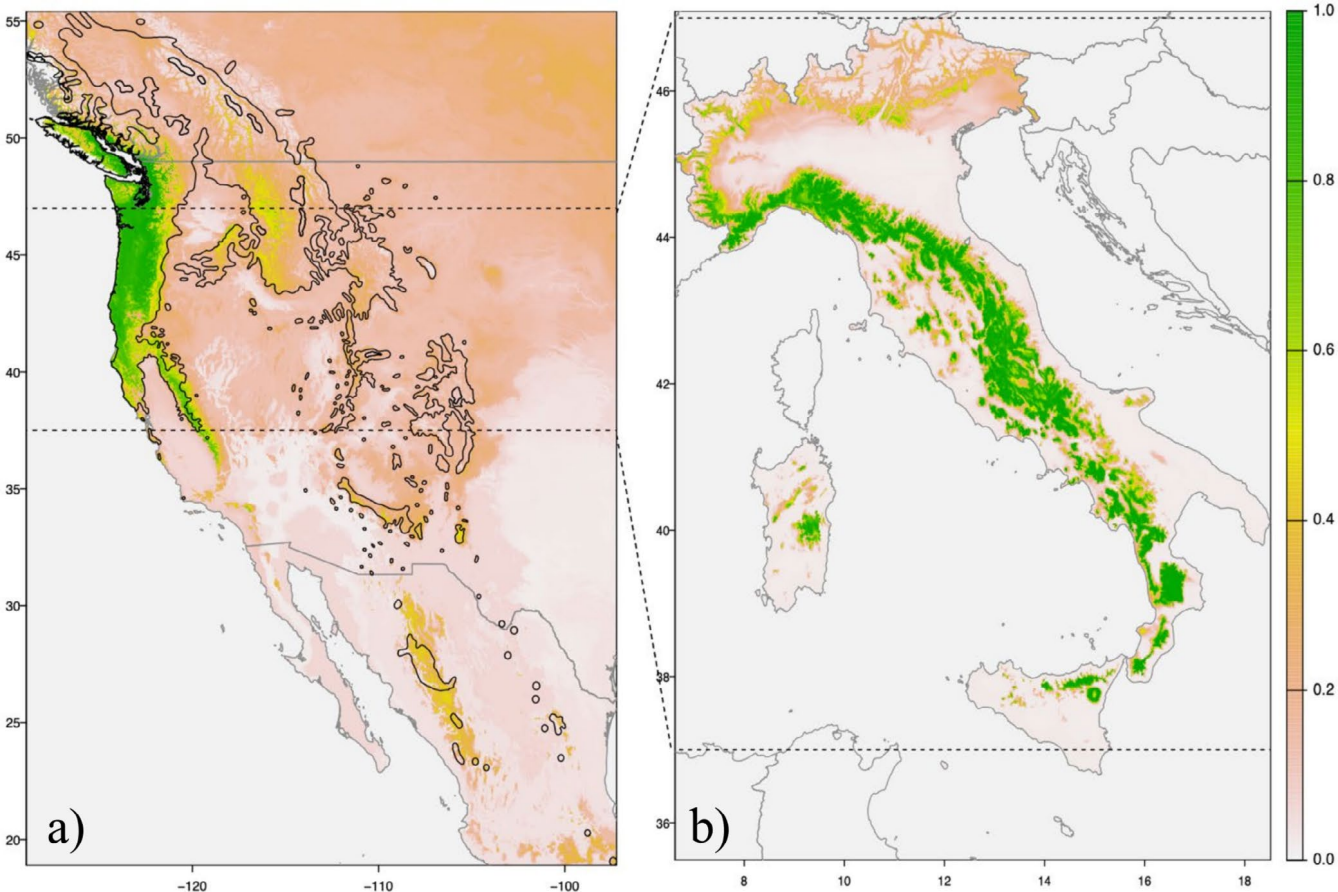
Spatial projection of the biomod2 ensemble model fitted on Italian Douglas‐fir plantations on: (a) the native range in the Pacific Northwest US, according to the 1901–1930 climatic normal period; and (b) in Italy, according to the current normal climate (1991–2020). The highest habitat suitability overlap (green colors) with the spatial occurrence in Italy was detected with environments covered by the coastal variety of the species. The dashed lines project the latitudinal range covered by Italy in the species' native range.

To identify suitable habitats for Douglas‐fir in Italy, projections were derived from the best‐fitting ensemble models, using the mean probability of survival weighted on the above‐mentioned fitting metrics. Other projection methods, such as mean, median, coefficient of variation, confidence intervals, and the average of binary predictions, yielded similar results and will not be discussed here. The developed model was tested under the climatic conditions in the species' native range (Northwestern America) to verify its reliability. The model's predictions showed a good match to the native region of the Douglas‐fir coastal variety (Figure 4a), thus confirming its robustness and accuracy. The predicted habitat suitability in Italy is shown in Figure 4b. The highest probability of survival was found for the Apennine chain and the western part of the Alps. In contrast, the lower probability of success for Douglas‐fir plantations was observed in lowlands and Southern Italy (including Sicily and Sardinia), except in high‐elevation areas, which showed a higher likelihood of survival. Indeed, the southernmost regions of Italy are characterized by environmental conditions that differ significantly from those used for modeling. In northern Italy, the high‐elevation areas of the Alps and the Dolomites, along with the continental inner valleys of the Alpine chain and the Po Valley, are unsuitable for the species. Likewise, the entire coastal area in southern Italy, characterized by a warm Mediterranean climate, was predicted to be unsuitable for Douglas‐fir survival, including the Apulia region and the low‐elevation areas of Calabria and Sicily.

**FIGURE 5:**
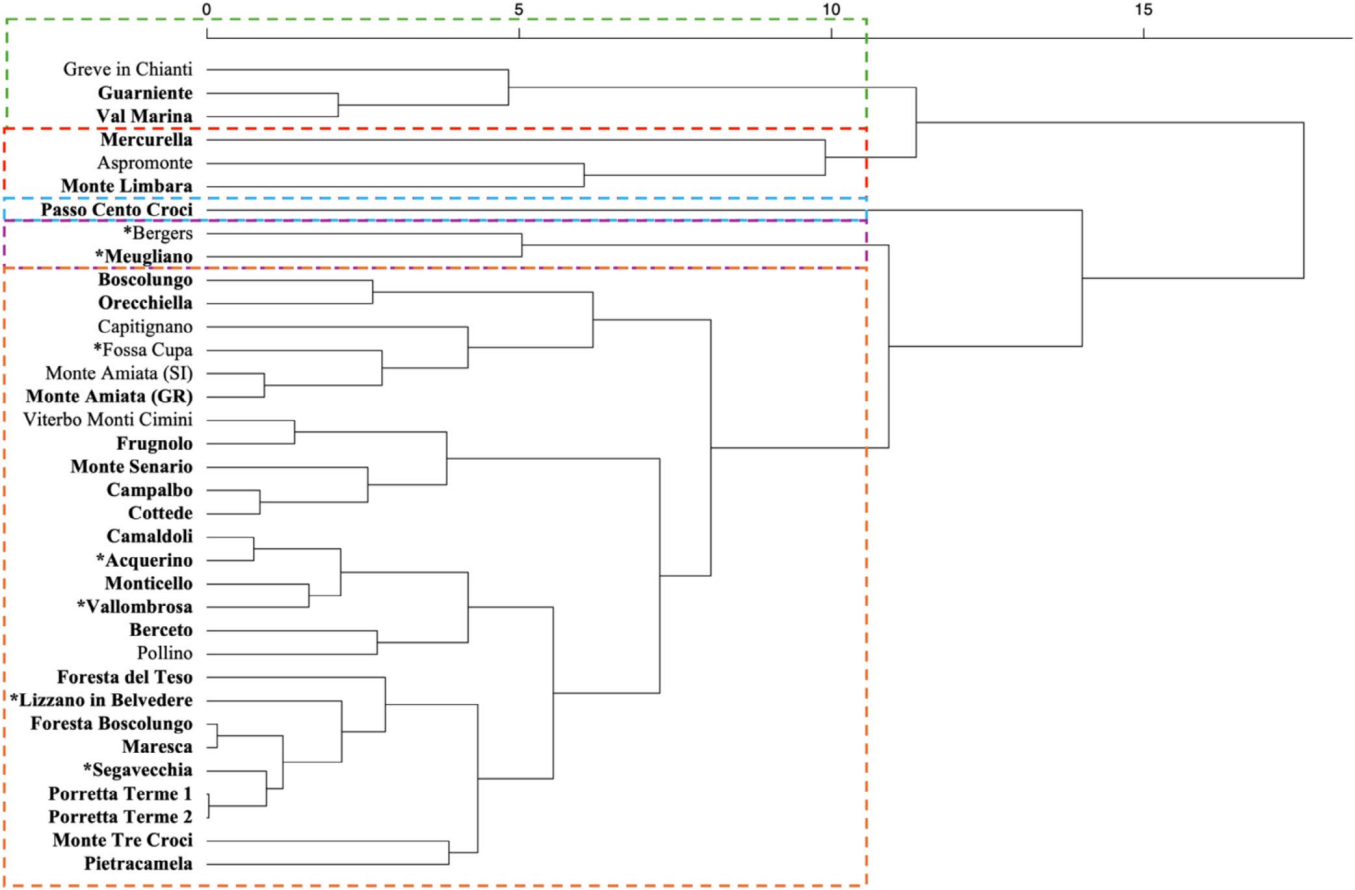
Results of the cluster analysis showing the climatic proximity of 35 Douglas‐fir stands spread over Italy. Five main groups of stands were identified, each represented by a different color box. The largest cluster (orange box) mainly includes stands distributed along the Apennine chain, while the purple box encompasses the stands located in Piedmont. The blue box contains only one plantation from Liguria, while the red one represents the southernmost plantations in the Italian range. The green cluster comprises plantations in central Italy at lower elevations. The 27 old‐growth stands (> 90 years old) are in bold, while the eight younger stands (< 70 years old) of unknown origin are in plain text. The seed stands included in the Italian National Register of Forest Reproductive Materials (FRM) are marked with an asterisk (*).

The cluster analysis was conducted on a subset of 35 Douglas‐fir stand sites chosen for their representativeness of the diverse ecological conditions across the Italian distribution of the species. Among the 35 stands selected, 27 were old‐growth (> 90 years), while 8 were younger (< 65 years old), as established on abandoned lands after the Second World War. The results showed that the different locations could be grouped into 5 main clusters (Figure 5), which were partly related to site latitude and elevation. The smallest group comprises the high‐elevation sites from Calabria (e.g., Mercurella and Aspromonte) and a trial site in Sardinia (Monte Limbara), while the largest group can be further divided into five additional sub‐clusters. As expected, two sites from Piedmont (Meugliano and Bergers, northwestern Italy) grouped together, while the location of Passo Cento Croci (northern Apennines) diverged from all the other sites due to its distinct ecological signature. Finally, the largest cluster comprises sites along the northern Apennines, between Tuscany and Emilia‐Romagna, where most of Pavari's experimental sites are located.

## 4 Discussion

The middles fitted on the experimental network showed the temperature regime at planting sites, expressed by GDD5, as the main factor affecting Douglas‐fir survival in Italy. Conversely and surprisingly, no clear evidence of species‐specific effects of the Mediterranean climate was noticeable, with AHM being less relevant than GDD5 in the models. Additionally, CMD and SHM were either poorly or not relevant even if often valuable when modeling tree species in the Mediterranean climates (Benito Garzón et al. 2019; Hallingbäck et al. 2021). This result was, however, somehow expected, since we were modeling an exotic tree species rather than a native one. A possible explanation for that can be found in previously published literature. Many authors reported that Douglas‐fir tolerates recurrent droughts but is susceptible to cold even in the planting range (Castaldi et al. 2020; Leuschner and Meinzer 2024; Thurm and Pretzsch 2021; Vangestel et al. 2018). In agreement with that, the most successful plantations in Italy were found in mild climates and mid‐elevation sites (650 m—1100 m a.s.l.) in Central and Southern Italy, which are characterized by a milder Mediterranean climate compared to those in the Alpine regions. This is consistent with the ecophysiological requirements of the species reported in the literature (Bansal et al. 2015; Eilmann et al. 2013; Vitali et al. 2017) and with findings from studies conducted in the native range (Lu et al. 2015) where it occurs across a vast geographical and elevational range. Moreover, it is worth noting that the bioclimate of the southern native range of the coastal variety (northern California and southern Oregon) is known to be very close to Mediterranean (Metzger et al. 2013). Accordingly, Douglas‐fir thrives in regions with relatively dry summers, mild and wet winters, and a long frost‐free period, with relatively slight diurnal temperature variations (6°C–8°C) and rainfall peaks during winter (Bansal et al. 2015; Griesbauer and Green 2010). However, Frei et al. (2022) suggested that Douglas‐fir can be favored in relatively dry, nutrient‐deficient areas where fast‐growing deciduous trees are limited by nutrient availability.

Spatial modeling assumes that the species distribution is in equilibrium with its environment (Araújo et al. 2005; Márcia Barbosa et al. 2013). Considering that trees can survive for long periods (some of our plantations exceed 100 years) and the rapid changes in climate since the 1960s, the outputs from our SDMs may carry a non‐negligible level of uncertainty (Littell et al. 2011). While accurate climatological time series and tailored climatic surfaces may partially address this issue, projections should be considered with caution, particularly for long‐living forest tree species. However, this also represents a risk for native trees currently used in plantations (Ray et al. 2025). In addition, the complexity of some “black‐box” algorithms, like RF and MaxEnt, must be carefully considered. These models utilize vast amounts of information as input data and have a complex internal structure, which can affect the importance of predictors and the internal balance among various components. For this reason, establishing a well‐distributed and spatially balanced network of common garden experiments is crucial in ecological modeling (Chakraborty et al. 2018; Hallingbäck et al. 2021). This is even more important when non‐native species are tested in new environments, such as Douglas‐fir in Europe.

### 4.1 Spatial Prediction and Survival of Douglas‐Fir Across Italy

The models developed in this study suggest that Douglas‐fir could potentially be utilized in afforestation and reforestation activities throughout Italy. However, flaws or warps in the distribution of suitable sites across the country may stem from the unequal placement of examined locations along Italy's climatic and environmental gradients, resulting from the lack of an appropriate spatial design for the experimental trials. In general, our dataset includes numerous surviving Douglas‐fir stands along the Apennines, the Calabrian regions, and Southern Italy. In contrast, several plots formerly established in the Alps failed in the 1990s, and Douglas‐fir plantations have not been re‐established there since. Notably, the Italian Alpine region exhibits a continental climate, such as Austria and Germany, where, in contrast, Douglas‐fir is widely cultivated. (Neophytou et al. 2019).

In this study, we found that only two parameters (GDD5 and AHM) are significant parameters of the model, while CMD, SHM, or TD are less meaningful for Douglas‐fir survival, in contrast to some literature results (Chakraborty et al. 2018; Isaac‐Renton et al. 2014). Surprisingly, the relative importance of CMD in the Mediterranean was much lower than expected. However, these results can be partially attributed to issues related to the unbalanced distribution of Douglas‐fir plantations in Italy. A large proportion of records in our spatial dataset is from typical Mediterranean areas, ranging from Tuscany to Calabria, at elevations between 800 and 1200 m a.s.l. In contrast, very few records originated from Northern Italy, where the local climate is harsher and characterized by snow, frost, and/or more abundant rainfall. Our model accurately mapped a large portion of the Douglas‐fir's native distribution range in the Northwestern US. This supports the working hypothesis that most Italian Douglas‐fir stands have been established using propagation material from this part of the species' native range. Indeed, the coastal variety of Douglas‐fir, which is acknowledged to be more resistant to drought, is presumed to be prevalent in Italy (De Rogatis et al. 2024; Marchi and Cocozza 2021) and more suitable for the Mediterranean climate. Further research efforts and new experimental plantations are needed to clarify the above issue.

When reprojected in the native range and in the past climate, the fitted SDM we developed for Douglas‐fit in Italy, showed a good habitat suitability overlap with the coastal part of the native range. This reverse projection was therefore confirming the climate matching between the two geographic areas and time slices (1901–1930 for North America and 1991–2020 for Italy) and the goodness of fit of this work. On the other hand, we recommend caution in interpretation because climate matching does not automatically imply that seeds were arriving from that part of the native range. Only molecular data may be able to answer the question “where seeds came from?” and new analysis should be performed (Hintsteiner et al. 2018; Neophytou et al. 2019; van Loo et al. 2015, 2019). However, it was demonstrated that, on average, all the modeling algorithms employed in this study demonstrated similar predictive performances for Douglas‐fir survival across Italy. Very small differences in all the used metrics were recorded between algorithms, which were all tuned to perform best. However, even if not much different, it is worth mentioning that RF‐based models, machine‐learning algorithms, and AI‐based solutions, in general, do not provide any confidence intervals associated with predicted values nor the model's coefficients. Confidence intervals are essential for researchers when running parametric models (Wesselkamp et al. 2024). They provide valuable indications about the accuracy and reliability of predictions, enabling a more informed interpretation of the results. Moreover, confidence intervals can help establish new experimental sites, such as common gardens and arboretums, in areas where predictions are less certain. This can help focus models with a broader ecological scope, ultimately leading to more reliable data on the adaptability, resilience, and plasticity of forest tree species and their origin (Fady and Rihm 2022). Given the above considerations, we believe that parametric models should be preferred over “black‐box” and AI‐based solutions, and that ensemble models can only partially address this issue.

### 4.2 The Potential Mismatch Between Genotypes and Local Climate

A key point concerning forest reproductive materials and forest genetic resources is the appropriate selection of provenances by matching the climate of origin with the target climate at the planting site (Aitken et al. 2008; Williams and Dumroese 2013; Yu et al. 2022). The primary limitation of this study is the unknown origin of the Douglas‐fir stands analyzed, as well as their unknown genetic makeup (Marchi and Cocozza 2021). Pavari's experimental work was based on the limited knowledge available at that time, which included minimal information about the seed sources used. Since that time, the best‐performing stands across Italy may have been utilized as new sources of propagation material for new plantations (Avolio and Bernardini 2000). This may further complicate the reconstruction of Douglas‐fir provenances used in Italy, emphasizing the need for comprehensive genetic studies on current populations in the country.

A thorough genetic characterization of the current Italian stands could help reconstruct the origin of the propagation material used, which is crucial for understanding the ecological behavior of the species outside its native range (Zhao and Wang 2023), as well as determining whether the broad adaptability of the species depends on the genetic makeup of the populations used or on their phenotypic plasticity. For instance, Vangestel et al. (2018) conducted association mapping and landscape genomics studies, integrating genotypic, environmental, and phenotypic data to disentangle the basis of cold‐hardiness adaptation in coastal Douglas‐fir. Furthermore, genetic characterization and modeling are crucial for predicting the most suitable varieties to mitigate the impacts of climate change (Isaac‐Renton et al. 2014).

The ecological relationships among the Douglas‐fir stands investigated in the cluster analysis do not necessarily reflect their geographic proximity, nor does geographic proximity reflect ecological relationships, as the same provenance could survive in two distant and distinct environments. In this regard, provenance tests are essential for understanding and predicting which seed sources are best adapted to future climatic conditions (Mihai et al. 2022). Knowledge of provenances' ecological niche is crucial, as the phenotypic plasticity could be insufficient for adapting to new environmental conditions. This may explain why Douglas‐fir failed in some of the studied plots (Chen et al. 2010). Further, adaptation to climate change could also be driven by epigenetic or regulatory effects, potentially impacting phenotypic responses, although the genetic mechanisms responding to climate change effects are still not fully understood (Rellstab et al. 2021).

## 5 Conclusions

Douglas‐fir is a successful non‐native tree species that has been experimentally tested in Europe, including Mediterranean parts of Italy, and represents a promising option for afforestation and reforestation activities, especially in areas where plantations of other species currently suffer from biotic factors. In this study, we confirmed the species' sensitivity to frost, as indicated by the GDD5 parameter, and the suitability of the entire mountainous region of Italy where the species has survived since the beginning of the last century. The climate matching between the native range in the Pacific Northwest and the introduction range in Italy supports the available information regarding the planting material imported into Italy. Also, our results will help in planning the future selection of new Douglas‐fir populations from the native area to serve as seed sources in the context of a rapidly changing climate. New sources may be tested in new common garden experiments that could be planted in the geographic areas at the margins of the predicted suitable area, and new data could feed new models for next‐generation plantations in Italy.

## Author Contributions

**Maurizio Marchi:** conceptualization (lead), formal analysis (equal), funding acquisition (equal), investigation (equal), methodology (lead), software (lead), writing – original draft (equal), writing – review and editing (equal). **Martina Cocozza:** data curation (equal), formal analysis (equal), investigation (equal), writing – original draft (equal). **Gabriele Bucci:** investigation (equal), writing – review and editing (equal). **Paolo Iovieno:** conceptualization (equal), funding acquisition (equal), investigation (equal), writing – review and editing (equal).

## Conflicts of Interest

The authors declare no conflicts of interest.

## Data Availability

All data used in this manuscript are freely accessible from ZENODO: https://doi.org/10.5281/zenodo.16735367. The data used for modeling are stored as an excel file (“dbCONmod_41‐81‐14‐24.xlsx”) and the folder contains a text file (“README.txt”) where the Excel columns are described. The shared folder also includes the raw ClimateDT output.
